# Arsenic compound sensitizes homologous recombination proficient ovarian cancer to PARP inhibitors

**DOI:** 10.1038/s41420-021-00638-2

**Published:** 2021-09-22

**Authors:** Junfen Xu, Yuanming Shen, Conghui Wang, Sangsang Tang, Shiyuan Hong, Weiguo Lu, Xing Xie, Xiaodong Cheng

**Affiliations:** 1 Department of Gynecologic Oncology, Women’s Hospital, Zhejiang University School of Medicine, Hangzhou, 310006 China; 2 Zhejiang University School of Medicine, Hangzhou, 310058 China; 3 Institute of Life Sciences, Chongqing Medical University, Chongqing, 400016 China; 4 Center of Uterine Cancer Diagnosis & Therapy of Zhejiang Province, Hangzhou, 310006 Zhejiang China; 5 Cancer Center, Zhejiang University, Hangzhou, 310058 Zhejiang China

**Keywords:** Ovarian cancer, Targeted therapies

## Abstract

The poly(adenosine diphosphate-ribose) polymerase (PARP) inhibitors show survival benefits in ovarian cancer patients with *BRCA1/2* mutation or homologous recombination (HR) deficiency, but only limited efficacy in HR-proficient ones. Another drug, arsenic trioxide (ATO) or arsenic drug (RIF), exerts antitumor effects via inducing DNA damage. Here, we investigated the combined therapeutic effects of the PARP inhibitors and the arsenic compound in HR-proficient ovarian cancer. The combined treatment of niraparib, olaparib, or fluazolepali with ATO showed a significant suppression in tumor cell viability and colony formation. The drug treatment also induced synergistic inhibition of cell proliferation and DNA damage, and acceleration of cell apoptosis in two HR-proficient ovarian cancer cell lines SKOV3 and CAOV3, either by simultaneous or sequential administration. The mechanism underlying these synergistic effects were reflected by the significantly increased ratio of cleaved-PARP/total PARP and decreased ratio of p-AKT/total AKT. Consistently, the combination of olaparib with RIF synergistically reduced the tumor growth in mouse xenograft models. In conclusion, the arsenic compound greatly sensitizes HR-proficient ovarian cancer cells to the PARP inhibitors, and our findings provide an evidence for the clinical treatment development of this combination in HR-proficient ovarian cancer patients.

## Introduction

Epithelial ovarian cancer is the most fatal type of gynecological cancers worldwide [1–3]. About half of the cases harbor defects in homologous recombination (HR) DNA repair in response to double strand breaks (DSB), and the rest is defined as the HR-proficient type [4, 5]. The pathogenesis of the HR-deficient type ovarian cancer has been well described, but the ones for HR-proficient type are poorly characterized. For the HR-deficient ovarian cancer, recent studies have shown that the application of the poly(adenosine diphosphate-ribose) polymerase (PARP) inhibitors rapidly produce synthetic lethality in cells with HR deficiency (HRD) [6–8].

The roles of the PARP inhibitors in HR-deficient ovarian cancer have been fairly well studied. It has been reported that PARP participates DNA repair in response to single-strand breaks (SSB) [9, 10] and that silencing PARP by pharmacologic inhibitors leads to persistent DNA SSB [11], which consequently convert to DSB at the replication forks [12, 13]. The unrepaired DSB caused by the PARP inhibitors trigger apoptosis in ovarian cancer cells with defects in HR, which is one major machinery to repair DSB with high fidelity [14]. Although they have been applied to treat HR-deficient ovarian cancer, the PARP inhibitors have very limited benefits for HR-proficient ovarian cancer [5].

Arsenic has been applied to a variety of cancers including ovarian cancer [15]. One great example is arsenic-rich traditional Chinese medicine “Ai-Ling #1” with high efficacy to cure acute promyelocytic leukemia (APL) and other cancers [15–17]. Its active ingredient arsenic trioxide (ATO) has been approved by FDA for the frontline treatment of APL [18]. Notably, Zhang et al. [19] showed that the PARP-1 inhibitor 4AN could sensitize hepatocellular carcinoma HepG2 cells to ATO treatment via abrogation of G2/M checkpoint and suppression of DNA damage repair. Moreover, the oral arsenic formulation realgar-indigo naturalis formula (RIF) shows a similar clinical efficacy to intravenous ATO but with a better safety profile, which has been incorporated into the China APL management guidelines [20–22].

In this study, we tested the combination effects of the PARP inhibitors such as niraparib, olaparib, or fluazolepali, and ATO in HR-proficient SKOV3 and CAOV3 human ovarian cancer cells. Our findings shed the lights on attractive application of PARP inhibitor-Arsenic compound for the treatment of HR-proficient ovarian cancers.

## Results

### Identification of the differential sensitivities of HR-proficient ovarian cancer cell lines to PARP inhibitors and ATO

**Fig. 1:**
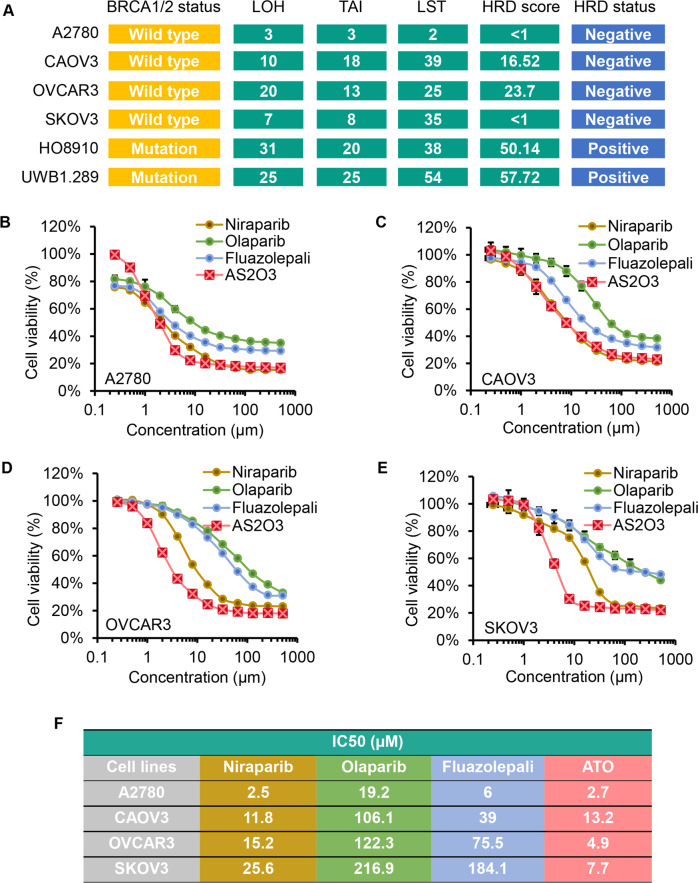
The sensitivity of HR-proficient ovarian cancer cell lines to PARP inhibitors and ATO. **A** BRCA1/2 and HRD status of selected ovarian cancer cell lines. **B–F** Drug-response curves of cell viability after PARP inhibitors (niraparib, olaparib, and fluazolepali) and ATO treatment, respectively, in HR-proficient ovarian cancer cells (A2780 (**B**), CAOV3 (**C**), OVCAR3 (**D**), and SKOV3 (**E**)) measured by CCK8 assay at 72 h. Mean ± SD shown (*n* = 3 biologically independent replicates per treatment and experiment repeated thrice). IC_50_ was calculated by GraphPad Prism 9.0 (**F**).

To determine the activities of the PARP inhibitors and ATO in the HR-proficient ovarian cancer cells, we assembled a panel of six ovarian cancer cell lines including A2780, CAOV3, OVCAR3, SKOV3, HO8910, and UWB1.289 cells, and selected for HR-proficient cells based on their *BRCA* status and HRD score analyzed by BGI DNA sequencing (Fig. 1A). The base line of HRD score is set on 30. The status is HRD-positive when HRD score is ≥30, whereas negative when the score is below 30. HRD scores of A2780, CAOV3, OVCAR3, and SKOV3 cells were <1, 16.52, 23.7, and <1, respectively, and these four cell lines are considered as HRD-negative. HO8910 and UWB1.289 cells carrying BRCA1/2 mutation are HRD-positive, both of which have HRD score greater than 50. Thus, the four HRD-negative cells were chosen as HR-proficient ovarian cancer cell lines for further study.

Three PARP1/2 inhibitors were tested in our studies. Among the three, niraparib and olaparib were approved by FDA, and fluazolepali was newly approved by National Medical Products Administration of China. To assess the relative sensitivity of PARP inhibitors and ATO, we assayed the cell viability in four HR-proficient ovarian cancer cells treated with each individual drugs (Fig. 1B–E). We found that the half maximal inhibitory concentration (IC50) of the three PARP inhibitors was much lower in A2780 cells than in the other three cell lines (Fig. 1F), which might be due to the differential expression of PTEN and BRAF. A2780 cells carry *PTEN* and *BRAF* mutation, while the other three lines harbor *PTEN* and *BRAF* wild type [23]. The IC50 of ATO was fairly lower in OVCAR3 cells, compared with CAOV3 or SKOV3 cells. Thus, we chose SKOV3 and CAOV3 cells, the relatively more resistant to all three PARP inhibitors or ATO, for further studies.

### Synergistic effects of PARP inhibitors and ATO in HR-proficient ovarian cancer cells

**Fig. 2:**
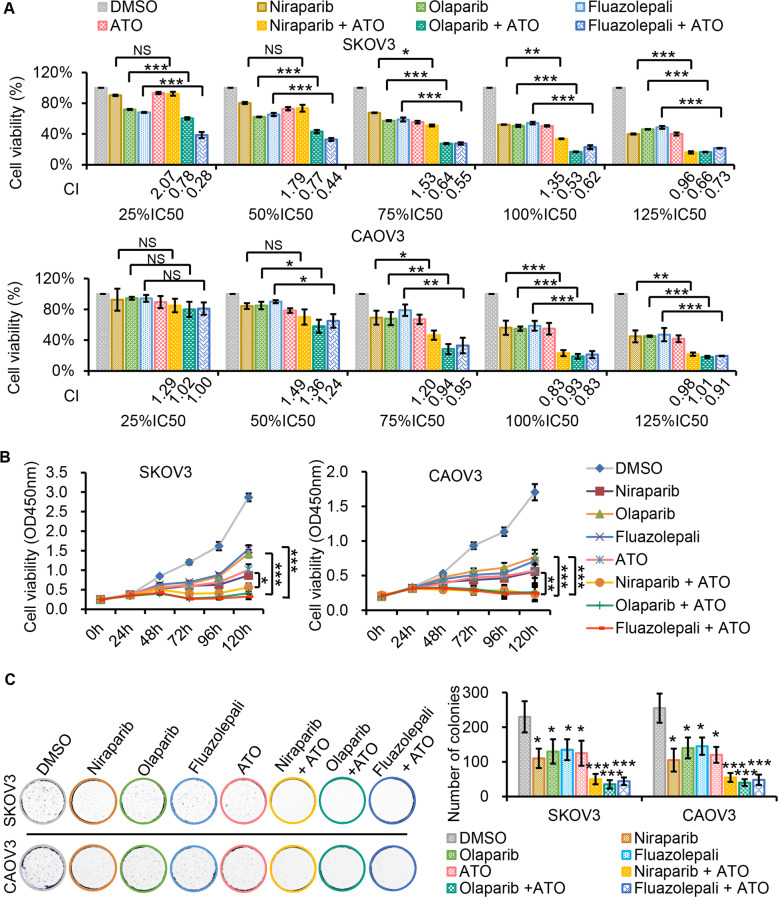
ATO-PARP inhibitor cotreatment decreases cell viability and colony formation. **A** Synergy analysis for ATO and PARP inhibitors, niraparib, olaparib, or fluazolepali, in HR-proficient SKOV3 and CAOV3 cells. Cells were treated with the indicated concentrations of ATO and niraparib or olaparib or fluazolepali for 72 h. Cell viability was detected by CCK8 assay. The combination index (CI) value was determined by CompuSyn software. CI value indicates the following: >1, antagonism; =1, additive effect; and <1, synergism. Bars indicate mean ± SD and are representative of three biological experiments. **B** Cells were treated with ATO or niraparib or olaparib or fluazolepali or ATO-niraparib or ATO-olaparib or ATO-fluazolepali using IC50 of each drug, and the cell viability was analyzed by time-lapse imaging for 120 h in the continued presence of drugs by CCK8 assay. Values represent the mean from three technical replicates. **C** Colony formation assays demonstrated synergy for the combination of ATO with niraparib or olaparib or fluazolepali in SKOV3 and CAOV3 cells. The concentration of these drugs used in this experiment was 10% of IC50. Representative images are shown of three independent experiments. Bars indicate mean ± SD and are representative of three biological experiments. *P < 0.05; **P < 0.01; ***P < 0.001.

The PARP inhibitors alone lack great effects on HR-proficient cancer. Here, we explored whether the add-on of PARP inhibitors and ATO has a synergistically apoptotic effect in HR-proficient ovarian cancer cells, since both the PARP inhibitors and ATO act to regulate DNA damage signaling. Figure 2A compared the cell viabilities of SKOV3 or CAOV3 cells treated with multiple set of drugs given at different IC50 doses (Fig. 2A). The synergetic effects of the drug combination were evaluated by the combination indexes (CIs) (Fig. 2A). For SKOV3 cells, the combination of olaparib or fluazolepali with ATO at 25, 50, 75, 100, or 125% of IC50s showed a significant collaboration on the cancer cell viabilities, given that the addition of ATO greatly reduced cell viabilities than the PARP inhibitors alone and that the according CI values were below 1. In contrast, the results for combination of niraparib and ATO were not conclusive since the synergistic effects (CI < 1) only occurred when the given dose was more than 100% of IC50s. Similar to the results observed in SKOV3 cells, the treatment of CAOV3 cells with olaparib or fluazolepali and ATO at 75 and 100% ratios of IC50s significantly sensitized cells than each itself individually. Niraparib and ATO showed a synergistic effect in CAOV3 cells at the 100 and 125% of IC50s. Overall, these results suggest that the addition of ATO sensitizes HR-proficient ovarian cancer cells to the PARP inhibitors, especially olaparib and fluazolepali, though the optimized efficacy appears at specific concentrations for each drug.

Next, we tested the cell viability inhibitory effects of ATO or the PARP inhibitors alone or the combination of both by Cell Counting Kit-8 (CCK8) assay (Fig. 2B). The cells were treated with different sets of drug conditions at 100% IC50 at various times. Indeed, both SKOV3 and CAOV3 cells exhibited increased sensitivities to the combination of ATO and niraparib, olaparib, or fluazolepali, compared to each alone. However, only olaparib or fluazolepali and ATO worked synergistically in both SKOV3 and CAOV3 cells, while the colony formation assays showed that the formed colonies of SKOV3 and CAOV3 cells were markedly reduced by all three combinations, probably due to the longer incubation time than the conditions in Fig. 2A, B (Fig. 2C).

### Combination of the PARP inhibitors and ATO increases DNA damage in HR-proficient ovarian cancer cells

**Fig. 3:**
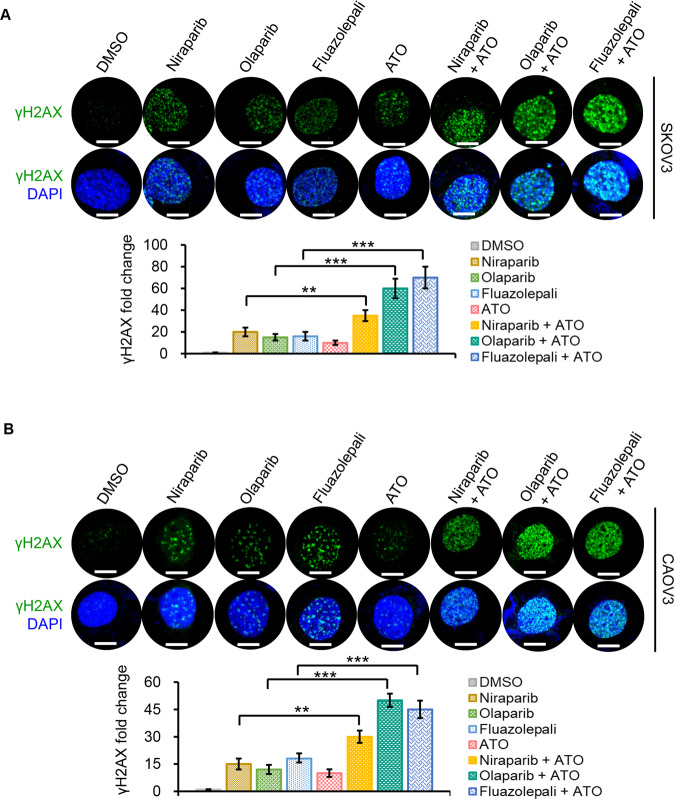
ATO and PARP inhibitors induce markers of DNA damage in SKOV3 and CAOV3 cells. SKOV3 (**A**) and CAOV3 (**B**) cells were treated with DMSO, ATO, niraparib, olaparib, fluazolepali, combination of ATO and niraparib, combination of ATO and olaparib, or the combination of ATO and fluazolepali using the IC50 of each drug for 48 h. Co-IF for γH2AX was performed. Cells were counterstained with DAPI. Representative images are shown of three biologically independent experiments. Magnification is ×60. Scale bar is 10 μm. Quantization of γH2AX expression was analyzed. The γH2AX fold change was evaluated by number of foci/cell and normalized with DMSO-treated group. A total of 50 cells from each slide were counted. Error bars represent mean ± SD. **P < 0.01; ***P < 0.001.

It is known that both the PARP inhibitors and ATO regulate DNA damage response. Here, we investigated whether the synergistic effects of the drug combination described above were due to changes of DNA damage response. To address this question, the effects on the accumulation of DNA DSB were studied in SKOV3 and CAOV3 cells treated with various drug combinations for 48 h. We examined the DNA DSB by monitoring DSB marker, γH2AX (Ser 139) by immunofluorescence. Both SKOV3 and CAOV3 cells accumulated more γH2AX expression when treated with the drug combination than that when treated with ATO, niraparib, olaparib, or fluazolepali alone (Fig. 3A, B). The immunofluorescent intensities of γH2AX expression under each condition were quantized in the bar figures accordingly.

To test whether ATO pretreatment would better improve the cell sensitivities to the PARP inhibitors, we added ATO 24 h before treatment with the PARP inhibitors. The γH2AX expression was significantly enhanced with sequential administration of ATO and the PARP inhibitors than the single agents in SKOV3 and CAOV3 cells (Fig. S1A, B). The DNA damage effects by this procedure were similar to that by the simultaneous treatment (Fig. 3A, B). Collectively, the combination of the PARP inhibitors and ATO result in accumulation of DNA DSB, suggesting that synergistic effect of the PARP inhibitors and ATO might be associated with enhanced DNA damage.

### The PARP inhibitors and ATO are synergistic in increasing apoptosis of SKOV3 and CAOV3 cells

**Fig. 4:**
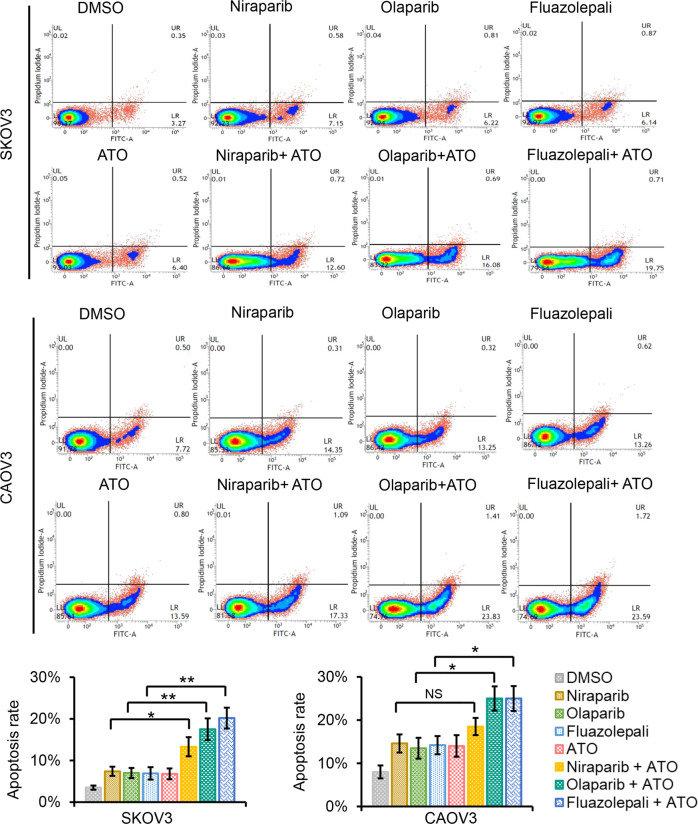
Cooperative treatment effects of ATO and PARP inhibitors on cell apoptosis in SKOV3 and CAOV3 cells. ATO and PARP inhibitors, niraparib, olaparib, or fluazolepali, cooperatively induced apoptosis of SKOV3 and CAOV3 cells. Cells were treated with DMSO, ATO, niraparib, olaparib, fluazolepali, combination of ATO and niraparib, combination of ATO and olaparib, or the combination of ATO and fluazolepali for 48 h, followed by FACS analysis of cell apoptosis with annexin V and PI staining. We used the IC50 of each drug for this experiment. Representative images of three biologically independent experiments. Error bars represent mean ± SD. NS not significant; *P < 0.05; **P < 0.01.

DNA damage is a trigger to cellular apoptosis. To test whether the combination of the PARP inhibitors and ATO accelerates cell death, we performed apoptosis analysis in the drug-treated SKOV3 and CAOV3 cells. The cell lines were monitored by flow cytometry using recombinant annexin V conjugated to green-fluorescent FITC dye to detect apoptotic cells and propidium iodide (PI) for dead cells. The PARP inhibitors or ATO individually demonstrated a slight increase of apoptosis, but the combination of ATO and olaparib or fluazolepali led to apoptosis at significantly higher levels (Fig. 4). In addition, combination of ATO and niraparib only modestly increased the percentage of apoptotic cells compared to the single drug treatment in SKOV3 and CAOV3 cells. Taken together, the PARP inhibitors combined with ATO induce cell early apoptotic signals in HR-proficient ovarian cancer cells.

### Combination of the PARP inhibitors and ATO decreases phospho-AKT (p-AKT) and cleaved-PARP expression

**Fig. 5:**
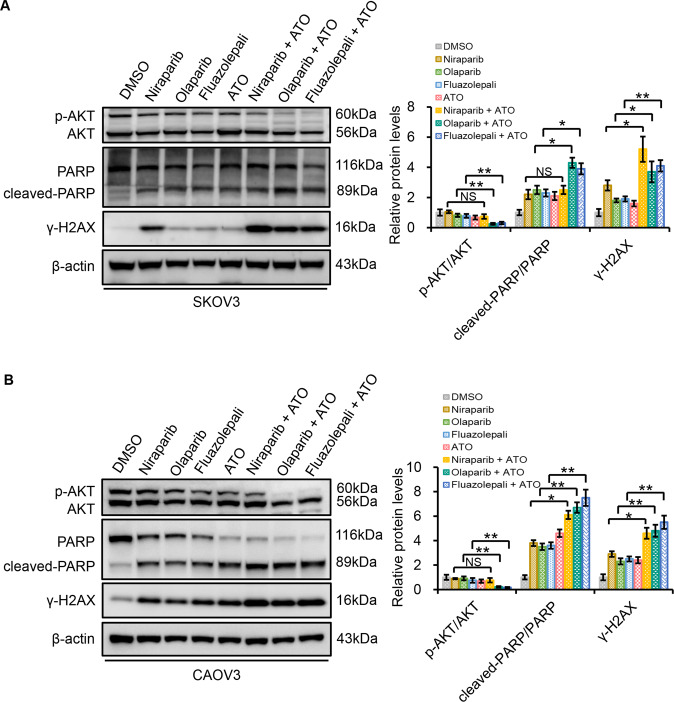
ATO cooperates with PARP inhibitors to induce cleaved-PARP and γH2AX expression and inhibits phosphor-AKT expression. SKOV3 (**A**) and CAOV3 (**B**) cells were treated with DMSO, ATO, niraparib, olaparib, fluazolepali, combination of ATO and niraparib, combination of ATO and olaparib, or the combination of ATO and fluazolepali for 72 h, followed by assaying total PARP, cleaved-PARP, total AKT, phosphor-AKT, and γH2AX by western blotting. β-actin loading as control. The shown blots are of samples derived from the same experiment. Representative of *n* = 3 biologically independent experiments. Error bars represent mean ± SD. NS not significant; *P < 0.05; **P < 0.01.

To further investigate how the drug combination leads to cell apoptosis as well as DSB in HR-proficient SKOV3 and CAOV3 cells, we investigated the role of AKT in these synergistic effects, which is known for regulating apoptosis. As shown in Fig. 5A, B, niraparib, olaparib, fluazolepali, or ATO alone could slightly suppress the protein levels of p-AKT. However, they did not significantly change the expression ratio of p-AKT/total AKT. In contrast, the combination of olaparib or fluazolepali with ATO greatly reduced the expression ratio of p-AKT/total AKT. Consistently, the expression ratio of cleaved-PARP/total PARP was modestly augmented in single drug-treated SKOV3 and CAOV3 cells, and largely induced in the cells treated with the combination of olaparib or fluazolepali with ATO. The effects were much stronger in ATO-olaparib and ATO- fluazolepali groups than those in ATO-niraparib group, consistent with our observation mentioned above in the Fig. 4. We also tested the expression of the DNA DSB marker γH2AX by western blot analysis. Consistent with the results detected by IF, western blot assay revealed the upregulation of γH2AX protein in SKOV3 and CAOV3 cells treated with ATO, niraparib, olaparib, or fluazolepali. The combination treatment significantly increased the expression of γH2AX protein as compared to single-agent treatment (Fig. 5A, B).

### RIF and olaparib are synergistic in suppressing the tumor growth of HR-proficient SKOV3-derived xenograft models

The tumor cells marked by luciferized SKOV3 (SKOV3-luc) were transplanted in mice intraperitoneally. The mice were then treated with vehicle (control), olaparib, RIF (the only commercially available oral arsenic drug), or a combination of olaparib and RIF. One pill of RIF is 270 mg, containing 125 mg of indigo naturalis, 50 mg of radix salviae miltiorrhizae, 45 mg of radix pseudostellariae, 30 mg of realgar, and 20 mg of garment film. For the PARP inhibitors, we selected the FDA-approved olaparib for animal study, because we found the much stronger inhibitory effects of olaparib with ATO than that of niraparib with ATO in vitro. Two doses of RIF, namely low dose of 135 mg/kg and high dose of 600 mg/kg, were tested for its synergistic effect with olaparib.

**Fig. 6:**
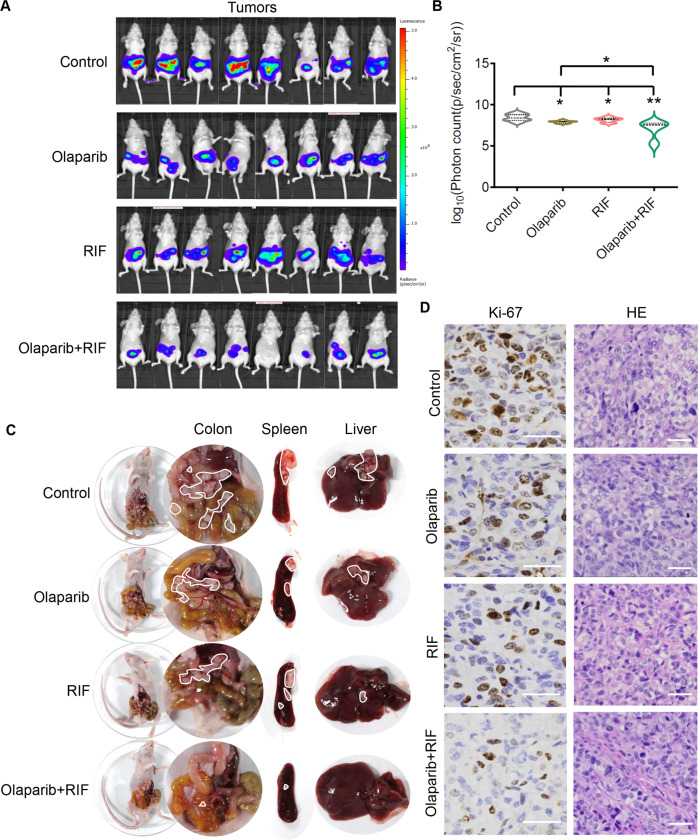
Low dose of RIF sensitizes SKOV3-derived xenograft tumors to olaparib in vivo. **A** Female BALB/c nude mice bearing luciferized SKOV3-derived tumors were randomized into four treatment groups and treated with vehicle control, low dose of RIF (135 mg/kg, daily by i.g.), olaparib (100 mg/kg, daily by i.g.), or RIF/olaparib combination for each model till 3 weeks (*n* = 8 mice per group). Tumor growth was monitored by weekly bioluminescence imaging of the mice. Representative images of mice bearing SKOV3 xenografts. **B** Tumor burden at the end of the treatment is represented as mean ± SD. **C** Representative images of the abdominal tumor nodules in colon, spleen, and liver in the four different treated groups are shown. **D** Tumors dissected from the SKOV3 xenografts with the indicated treatments were examined for Ki-67 protein expression by IHC and HE staining. Scale bar, 100 μm. *P < 0.05; **P < 0.01.

First, the mice were fed with low-dose RIF, 100 mg/kg of olaparib, or cotreatment of low-dose RIF and 100 mg/kg of olaparib every day for 3 weeks. We found that the cotreatment effectively reduced tumor growth, compared to RIF or olaparib alone (Fig. 6A, B). Importantly, RIF and olaparib cotreatment dramatically reduced the invasive potential of SKOV3-derived xenograft tumors by reducing the formation of abdominal tumor nodules in the colon, spleen, and liver, compared with that in single drug treatment group (Fig. 6C). Moreover, the cotreatment also downregulated the expression of Ki-67, a cell proliferation marker, in the xenograft tumors (Fig. 6D). In addition, the safety of RIF and olaparib was tested in heart, lung, kidney, liver, colon, and spleen of the 5-week female BALB/c nude mice. The BALB/c nude mice were directly administered orally each day with low-dose RIF, 100 mg/kg of olaparib, or the combination of RIF-olaparib. The administered mice were executed after 2 months and analyzed for HE staining of heart, lung, kidney, liver, colon, and spleen. No fatal toxicity of these organs was found (Fig. S2).

Secondly, high-dose RIF with olaparib were given to the tumor mice to determine whether there were better cooperative effects to suppress the tumor growth. We assessed tumor growth in response to daily treatment with high-dose RIF, olaparib, or high-dose RIF plus olaparib for 2 weeks. While we found that each drug treatment alone led to an inhibition in tumor growth compared to the control, the most significant suppression of tumor growth was achieved by the treatment of the combination of RIF plus olaparib, and the effect by high-dose RIF plus olaparib is stronger than the one of low-dose RIF plus olaparib (Fig. S3). Collectively, these in vivo findings highlight RIF plus olaparib as a promising alternative for current clinical therapy.

## Discussion

Our studies demonstrated the first evidence that combination of the PARP inhibitors and the arsenic compound, which individually have poor therapeutic effects for HR-proficient ovarian cancer, show a greatly synergistic impact on this specific type of ovarian cancer.

The PARP inhibitors are known for their great benefits for HR-deficient ovarian cancer but poorly for HR-proficient ovarian cancer. This leads scientists to seek possible combination of the PARP inhibitors and other drugs as new therapeutic approaches for HR-proficient ovarian cancer. We chose to combine ATO and the PARP inhibitors based on the facts that both participate regulation of DNA damage [24–28]. Our data in SKOV3 and CAOV3 HR-proficient ovarian cancer cell lines showed that ATO combination with all three PARP inhibitors, especially olaparib and fluazolepali, led to significant accumulation of DNA damage as well as cell apoptosis, promoting cancer cell death. Moreover, the treatment of the oral arsenic drug RIF and olaparib also displayed cooperative effects in inhibiting transplanted tumor growth and metastasis in mice.

Our studies suggest that the drug combination among the PARP inhibitors and ATO collaboratively act on DNA damage and cell apoptosis in HR-proficient ovarian cancer. Since it was reported that PARP inhibition could induce AKT alterations in a set of cancers [29–31], we tested the possibilities of whether AKT plays a critique role in the drug-induced DNA damage and cell apoptosis. In the HR-proficient cells, the cotreatment significantly upregulated the levels of cleaved-PARP and decreased the phosphor-AKT levels. Consequently, the drug combination significantly increased the levels of γH2AX expression. Consistently, Huang et al. reported that ATO treatment could cause AKT inactivation, followed by increased GSK3β-mediated MCL1 degradation and apoptosis in leukemia cells [32]. Another study showed that ATO leveled up the p53 protein expression and induced cleavage of PARP, with appearance of the 85 kDa cleavage product in human gastric cancer cells [28]. Taken together, our findings, along with those from previous studies [33], suggest that olaparib combined with ATO enhance inhibition of PI3K/AKT pathway, leading to phosphorylation of H2AX and induction of DNA damage in HR-proficient cells.

Altogether, our results herein demonstrate the synergistic effects of the PARP inhibitors and the arsenic compound in HR-proficient ovarian cancer cells in vitro and xenograft tumors derived from HR-proficient ovarian cancer cells in vivo, which do not respond well to the PARP inhibitors alone. Further, our findings provide an evidence for the clinical development of this combination in HR-proficient ovarian cancer patients.

## Materials and methods

### Cell lines

Human ovarian cancer cell lines CAOV3, OVCAR3, SKOV3, and UWB1.289 were purchased from the American Type Cell Culture. A2780 was purchased from Sigma (Cat# 93112519). HO8910 was purchased from National Collection of Authenticated Cell Cultures of China (Cat# TCHu 24). SKOV3 cells (Cat# HTB-77) were cultured in McCoy’s 5A (BasalMedia, L630KJ). UWB1.289 cells (Cat# CRL-2945) were grown in RPMI 1640 (Cellmax, CGM112.05) and MEGM Bullet Kit (Lonza, CC-3150) at a 1:1 ratio. CAOV3 (Cat# HTB-75) and OVCAR3 (Cat# HTB-161) were cultured in Dulbecco’s Modified Eagle’s Medium (BasalMedia, L110KJ). A2780 and HO8910 were grown in RPMI 1640 (BasalMedia, L210KJ). All culture media contained 10% fetal bovine serum (Gibco, 7471) and 1% penicillin/streptomycin (Invitrogen, 15140-122). The cells were determined to be mycoplasma-free and cultured at 37 °C with 5% CO_2_.

### Drug sensitivity assay and cell viability assay

Stock solutions of niraparib (Shanghai Zai Lab Co., Ltd; 100 mmol/L), olaparib (MedChemExpress, HY-10162; 100 mmol/L), and fluazolepali (Jiangsu Hengrui Medicine Co., Ltd; 100 mmol/L) were prepared in dimethyl sulfoxide (DMSO) (Sigma, D2660) and stored in aliquots at −80 °C. Ai-Ling #1 solution (Harbin Yida Pharmaceutical Co., Ltd; 1 mg/mL) was stored at room temperature. Appropriate dilutions were prepared in culture medium. For viability assays, 2000 cells per well were seeded in 96-well plates and treated with DMSO or graded concentrations of niraparib, olaparib, fluazolepali, ATO, or a combination of PARP inhibitor-ATO drugs for 72 or 120 h. Cell survival was determined by CCK8 assay (DOJINDO Laboratories, CK04). Cell viability was then calculated relative to DMSO-treated groups and dose–response curves or IC50 plots were generated using GraphPad Prism 9.0. Synergy between PARP inhibitor and ATO was calculated using CompuSyn software [34].

### Animal studies and ethical approval

A total of 48 female BALB/c nude mice, age 5 weeks, were purchased from the SLAC (Shanghai Slack Laboratory Animal Co., Ltd, Shanghai, China) and kept under standard recommended conditions in the animal research center of Zhejiang Chinese Medical University. For xenograft experiments, approximately 1 × 10^6^ luciferized ovarian cancer cells (SKOV3-luc) were mixed in 100 μL PBS, injected intraperitoneally into the nude mice, and randomized eight or four mice for each group in a blinded manner. Tumors were typically established 5–7 days after implantation. For the drug administration, the animals were injected orally with olaparib alone, RIF alone, and olaparib combined with RIF or control (PBS) daily for 3 or 2 weeks. Tumor burden was measured serially once per week by bioluminescence imaging using the In Vivo Imaging System Lumina LT system (PerkinElmer, USA). The bioluminescence value of the tumors in each mouse was analyzed by Living Image software (PerkinElmer, USA). Results were presented as means ± SD.

### Statistical analysis

Experiments were routinely performed using at least two biological replicates and independently repeated at least three times. GraphPad Prism 9.0 was used for statistical analyses. All data are presented as the means ± SD, followed by determining significant differences using the two tailed Student’s *t* test or one-way analysis of variance test, where *P < 0.05, **P < 0.01, and ***P < 0.001. Drug combinations between PARP inhibitor and ATO were analyzed using CompuSyn software [34]. CI value indicates the following: >1, antagonism; =1, additive effect; and <1, synergism.

## Supplementary information

Supplementary methods

Supplementary figure legends

Figure S1

Figure S2

Figure S3
