# Supplementary material for: Arsenic compound sensitizes homologous recombination proficient ovarian cancer to PARP inhibitors
Source: Cell Death Discov. 2021 Sep 22;7:259. doi: 10.1038/s41420-021-00638-2 (PMC8458481; doi:10.1038/s41420-021-00638-2)
Supplement: Supplementary file 1 — Supplementary methods [file 41420_2021_638_MOESM1_ESM.docx]

**Supplementary materials and methods**

**HRD detection**

The genomic DNA was extracted using TIANamp Genomic DNA kit (Tiangen, DP304-03). HRD detection was done by BGI, China. Briefly, the DNA was interrupted and end-repaired, and a linker with a tag sequence was added to both ends of the DNA by ligase, followed by PCR amplification to form a pre-hybridization library (pre-PCR library). The target DNA fragment in the library was hybridized with a biotin-labeled HRD probe containing 93,200 SNP sites which could anchor target DNA fragment on the avidin magnetic beads through the biotin affinity reaction. After purification, the enriched DNA was specifically captured and amplified by PCR to obtain a post-PCR library (post-PCR library). The post-PCR library undergoes single-strand separation, circularization and rolling circle replication to generate DNA nano balls, which were sequenced by a gene sequencer. The finally HRD score was obtained via raw data filtration, comparison, deduplication, quality control, BAF (B allele frequency) and LRR (Log R) calculation. ASGAD (Allele Specific Gene-scar Analysis tool for Diagnosis) is the core genomic scar analysis algorithm for HRD assay, which is patented by BGI. ASGAD can be used to measure the Loss of Heterozygosity (LOH), the Telomeric Allelic Imbalance (TAI), and the Large-scale State Transitions (LST) in gDNA isolated from tumor tissue specimens or tumor cell lines. Meanwhile, the differences in purity and ploidy of tumor cells are also taken into account in the algorithm. The HRD score = LOH + LST + TAI - 15.5 × ploid. HRD cutoff value was further determined by receiver operating characteristic (ROC) analysis. HRD score ≥30 indicates positive; HRD score < 30 indicates negative.

**Colony formation assay**

2,000 cells per well were seeded into 6-well plates and treated continuously with the inhibitors for 10 days for colony growth. Once colonies had developed the cells were fixed in 70% ethanol for 10 min and stained with 2% crystal violet solution (Sigma, C6158) for 5 min. Plates were then imaged with a camera (Canon).

**Cellular apoptosis assays**

Cells treated as indicated were harvested, washed in PBS, and then resuspended in binding buffer containing annexin V-fluorescein isothiocyanate (FITC) and PI (Multi Sciences, AP101) for 15 min in the dark at room temperature. The apoptosis rate was measured on a BD flow cytometer (BD Biosciences, FACSVerse).

**Immunofluorescence**

Cells were plated onto autoclaved coverslips 24 hours prior to drug treatment. Cell were fixed in 4% paraformaldehyde (Servicebio, G1101) for 15 minutes at room temperature, washed with PBS, and then permeabilized with PBS-T (PBS, 0.3% Triton X-100) for 5 minutes. After blocking with 10% FBS containing 0.3% Triton X-100 (VETEC, V900502) for 1 hour, cells were incubated with primary antibodies (γH2AX (Abcam, ab26350) 1:5000) for 1 hour at room temperature. Coverslips were washed three times in PBS-T and stained with the appropriate fluorescently conjugated secondary antibodies (goat anti-mouse IgG (H+L) Alexa Fluor Plus-488 labeled secondary antibody (Invitrogen, A32723) 1:1000) for 1 hour at room temperature. Coverslips were washed in PBS-T and then mounted with a solution containing DAPI (Abcam, ab104139) and fluorescence photos were finally taken using a confocal laser-scanning microscope (Olympus, FLUOVIEW FV1200).

**Western blotting**

Treated cells were lysed by RIPA (Beyotime, P0013B) with protease inhibitor PMSF (Cell Signaling Technology, 8553S) and phosphatase inhibitor (Bimake, B15001). The protein concentration was quantified using a BCA protein assay kit (Fdbio science, FD2001), and equivalent amounts of total protein were loaded for experiment. Western blots were performed following a standard procedure as previously described [1]. Briefly, it was performed with the following primary antibodies: gamma H2AX (phospho S139) (Abcam, ab26350), AKT (Abcam, ab179463), phospho‑AKT (Ser 473) (Cell Signaling Technology, 4060), PARP (Cell Signaling Technology, 9542), and β-actin (Proteintech, 66009).

**Immunohistochemistry (IHC)**

IHC was performed on paraffin-embedded ovarian tumor xenografts with the use of primary antibody to Ki-67 (Cell Signaling Technology, 9449) as previously described [1]. All section slides were detected by standard hematoxylin and eosin (HE) staining.

**References**

1. Xu J, Lu W. FAM83A exerts tumorsuppressive roles in cervical cancer by regulating integrins. International journal of oncology. 2020; 57:509-521.
