# Supplementary material for: Arsenic compound sensitizes homologous recombination proficient ovarian cancer to PARP inhibitors
Source: Cell Death Discov. 2021 Sep 22;7:259. doi: 10.1038/s41420-021-00638-2 (PMC8458481; doi:10.1038/s41420-021-00638-2)
Supplement: Supplementary file 2 — Supplementary figure legends [file 41420_2021_638_MOESM2_ESM.docx]

**Figure S1** Drug effects of sequential administration of ATO and PARP inhibitors on DNA double-strand breaks. A and B, SKOV3 (a) and CAOV3 (b) cells were pre-treated with ATO for 24 hours, and then co-treated with niraparib, olaparib or fluazolepali for further 48 hours. Co-IF for γH2AX was performed. Representative images and quantification of γH2AX expression. Cells were stained for DAPI to label nuclei. Magnification is ×60. Scale bar, 10 μm. Error bars represent mean ± SD. **P*< 0.05；*** *P*< 0.001.

**Figure S2** The toxicity of the combination of RIF and olaparib *in vivo*. Female BALB/c nude mice were randomized into three different treatment groups and treated with low dose of RIF (135 mg/kg, daily by i.g.), olaparib (100 mg/kg, daily by i.g.), or RIF/olaparib combination for two months. A, Representative images of mice organs, including heart, lung, kidney, liver, colon, and spleen in each group at the end of the treatment. B, Representative images of HE staining of each organ in three treated groups. Scale bar = 10 μm.

**Figure S3** The efficacy of combining high dose of RIF and olaparib in SKOV3 xenograft models. Luciferized SKOV3 cells were intraperitoneally injected into the nude mice. Randomized mice were treated with vehicle control, high dose of RIF (600 mg/kg, daily by i.g.), olaparib (600 mg/kg, daily by i.g.), or a combination for each model till two weeks (n = 4 mice per group). At the end of treatment, the mice were euthanized. A, Tumor growth was monitored by bioluminescence imaging of the mice. Representative images of mice bearing SKOV3 xenografts from the indicated treatment groups. B, Tumor burden in four treated groups is represented as mean ± SD. * *P*< 0.05；***P*< 0.01.
