# Supplementary figures and images for: Arsenic compound sensitizes homologous recombination proficient ovarian cancer to PARP inhibitors
Source: Cell Death Discov. 2021 Sep 22;7:259. doi: 10.1038/s41420-021-00638-2 (PMC8458481; doi:10.1038/s41420-021-00638-2)

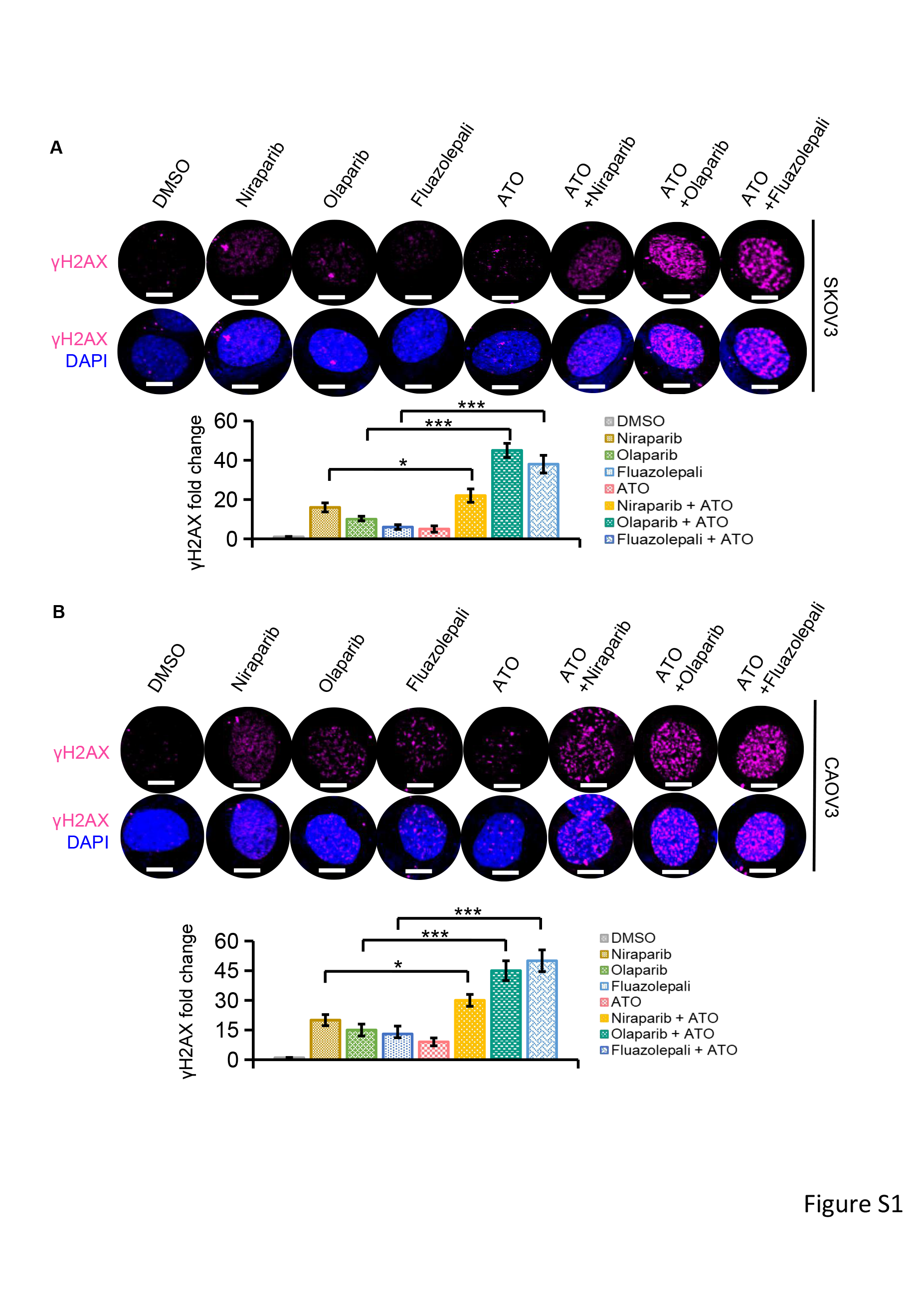

Supplement: Supplementary file 3 — Figure S1 [file 41420_2021_638_MOESM3_ESM.tif]

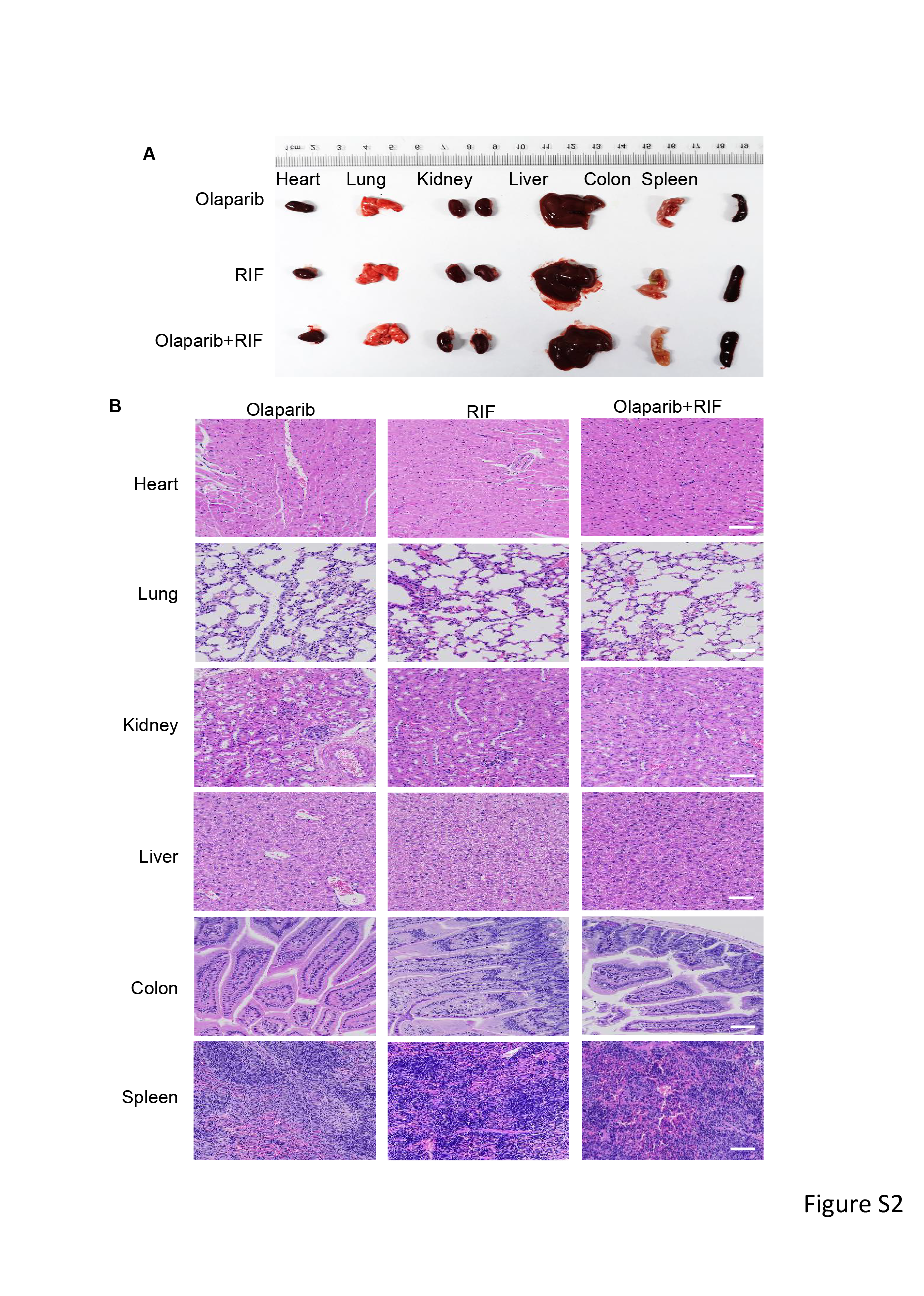

Supplement: Supplementary file 4 — Figure S2 [file 41420_2021_638_MOESM4_ESM.tif]

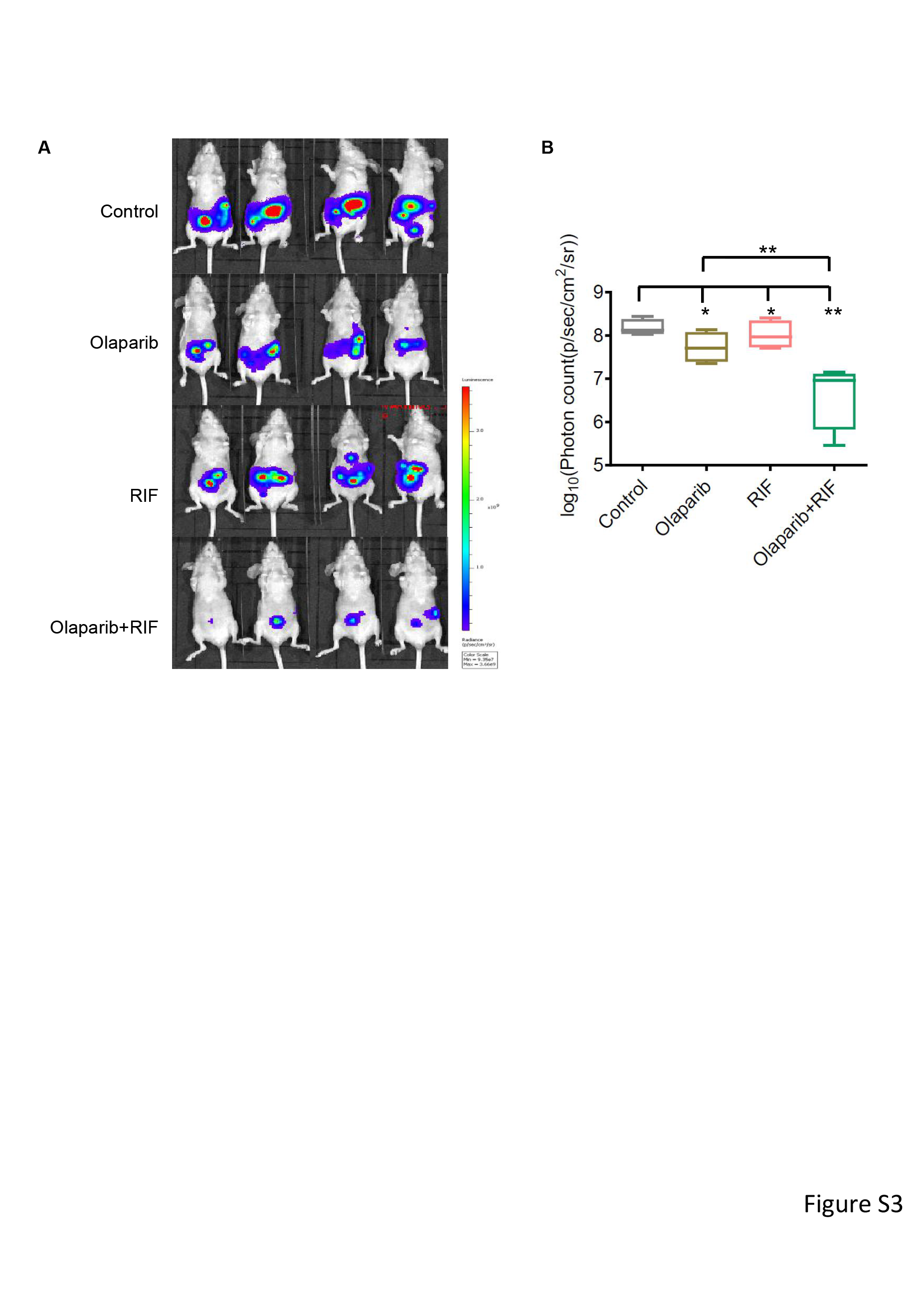

Supplement: Supplementary file 5 — Figure S3 [file 41420_2021_638_MOESM5_ESM.tif]
